# A novel class of peptide pheromone precursors in ascomycetous fungi

**DOI:** 10.1111/j.1365-2958.2010.07295.x

**Published:** 2010-07-30

**Authors:** Monika Schmoll, Christian Seibel, Doris Tisch, Marcel Dorrer, Christian P Kubicek

**Affiliations:** Research Area Gene Technology and Applied Biochemistry, Institute of Chemical Engineering, Vienna University of TechnologyGetreidemarkt 9/1665, Vienna 1060, Austria

## Abstract

Recently, sexual development in the heterothallic ascomycete *Trichoderma reesei* (anamorph of *Hypocrea jecorina*) has been achieved and thus initiated attempts to elucidate regulation and determinants of this process. While the α-type pheromone of this fungus fits the consensus known from other fungi, the assumed a-type peptide pheromone precursor shows remarkably unusual characteristics: it comprises three copies of the motif (LI)GC(TS)VM thus constituting a CAAX domain at the C-terminus and two Kex2-protease sites. This structure shares characteristics of both a- and α-type peptide pheromone precursors. Presence of hybrid-type peptide pheromone precursor 1 (*hpp1*) is essential for male fertility, thus indicating its functionality as a peptide pheromone precursor, while its phosphorylation site is not relevant for this process. However, sexual development in a female fertile background is not perturbed in the absence of *hpp1*, which rules out a higher order function in this process. Open reading frames encoding proteins with similar characteristics to HPP1 were also found in *Fusarium* spp., of which *Fusarium solani* still retains a putative a-factor-like protein, but so far in no other fungal genome available. We therefore propose the novel class of h-type (hybrid) peptide pheromone precursors with *H. jecorina* HPP1 as the first member of this class.

## Introduction

The mating process in fungi has been subject to elaborate studies for decades (Debuchy and Turgeon, 2006; Aanen and Hoekstra, 2007; Debuchy *et al*., 2010). Communication between haploid cells of opposite mating type is thereby crucial for the initiation of the mating process in heterothallic fungi. This interaction is predominantly achieved by the secretion of the small, diffusible peptide pheromones, which can act at a distance and relate their signal via specific receptors (Bolker and Kahmann, 1993; Kothe, 1999). Presently, two types of pheromone precursor genes have been identified in fungi, which are related to the MFa and MFα genes of *Saccharomyces cerevisiae* representing the best-studied examples (Kurjan, 1993; Wang and Dohlman, 2004).

Pheromone signalling causes directed growth of the trichogyne, the female organ, towards the male cells opposite mating-type in heterothallic fungi (Bistis, 1981; 1983;). The respective pheromone precursor genes were isolated from several heterothallic filamentous ascomycetes including *Cryphonectria parasitica*, *Magnaporthe grisea, Neurospora crassa* and *Podospora anserina* (Zhang *et al*., 1998; Shen *et al*., 1999; Bobrowicz *et al*., 2002; Coppin *et al*., 2005). In those ascomycetes, one of the precursor genes encodes a polypeptide containing multiple repeats of a putative pheromone sequence bordered by protease processing sites. These resemble the α-factor precursor gene of *S. cerevisiae* and the P-factor precursor gene of *Schizosaccharomyces pombe* respectively (Singh *et al*., 1983; Imai and Yamamoto, 1994). The other gene encodes the precursor for a short polypeptide with a C-terminal CaaX motif expected to produce a mature pheromone with a C-terminal carboxy methyl isoprenylated cysteine. Structurally similar pheromones were not only found in *S. cerevisiae* a-strains, *S. pombe* M-strains and numerous ascomycetes, but also in basidiomycetes (Michaelis and Herskowitz, 1988; Davey, 1992; Spellig *et al*., 1994; Olesnicky *et al*., 1999). In addition, the pheromone precursor genes are expressed in a mating-type-specific manner in *N. crassa, C. parasitica, P. anserina* and *M. grisea* (Zhang *et al*., 1998; Shen *et al*., 1999; Bobrowicz *et al*., 2002; Coppin *et al*., 2005). Pheromones have been shown to be essential for male fertility (Coppin *et al*., 2005). In *C. parasitica*, which contains two a-type peptide pheromone precursor genes (Zhang *et al*., 1993; 1998;), deletion of one of them does not prevent fertilization, but resulted in barren perithecia and defects in asexual sporulation. However, the defects in asexual sporulation may also be due to additional small open reading frames (ORFs; only one of which represents the a-type peptide pheromone precursor gene), in the described deletion strain (Turina *et al*., 2003). In contrast, it was reported that peptide pheromones are dispensable for female functions during sexual reproduction (Coppin *et al*., 2005; Kim and Borkovich, 2006). In *N. crassa*, the appropriate pheromone receptor (Kim and Borkovich, 2004), the heterotrimeric G–α-subunit GNA1 (Ivey *et al*., 1996) and both the G-protein β- and γ-subunits (Krystofova and Borkovich, 2005; 2006;) are necessary for chemotropic behaviour and perithecial development in females.

Although it seemed clear that the major role of pheromones in filamentous heterothallic ascomycetes is the control of recognition between male and female organs of opposite mating types, both types of pheromone precursor genes have also been identified in the homothallic ascomycetes *Sordaria macrospora* (Poggeler, 2000) and *Gibberella zeae* (Kim *et al*., 2008; Lee *et al*., 2008). Hence, the peptide pheromones and/or their precursor proteins may also have functions beyond attracting a mate. In some fungi, pheromones play a role in post-fusion events as well as cell–cell recognition and fusion. Specifically, pheromones have been implicated in the induction of meiosis in *S. pombe* (Willer *et al*., 1995; Chikashige *et al*., 1997) and stimulation of filamentous growth in *Ustilago maydis* (Spellig *et al*., 1994).

For more than 50 years, *Trichoderma reesei* was known only from one single isolate (QM6a), which was collected as an agent deteriorating cotton material of the US Army in the South Pacific during the World War II. Due to its ability to produce high amounts of cellulases, it served as the sole progenitor of many mutants that have been used for cellulase production thereafter (El-Gogary *et al*., 1989). Using molecular diagnostic methods, however, *T. reesei* was subsequently recognized to be indistinguishable from the tropical ascomycete *Hypocrea jecorina* (Kuhls *et al*., 1996). Nevertheless, attempts to induce mating of the original isolate QM6a or its industrially applied progeny had failed thereafter and *T. reesei* was consequently suggested to be an asexually propagating clonal line of the genus. We recently achieved induction of sexual development of *T. reesei/H. jecorina* QM6a, which is a MAT1-2 strain, with the MAT1-1 mating type of wild-type isolate *H. jecorina* CBS999.97 and thus disproved this assumption (Seidl *et al*., 2009). Upon mating of these strains fertilized stromata and mature ascospores are formed. However, this study also revealed that QM6a, while being male fertile, is female sterile. The identification of a sexual breeding system for this fungus is of utmost importance for strain improvement and initiated more detailed studies on the mechanism of sexual development in *T. reesei/H. jecorina*. However, while industrial applications of *T. reesei* and the enzymes produced by this fungus are well studied (Kubicek *et al*., 2009; Schuster and Schmoll, 2010), research towards elucidation of biology of sexual development is only in its beginnings and reproductive structures as well as the precise mechanism of fertilization remain to be studied.

Here, we report the finding of a protein resembling an a-type peptide pheromone precursor, which also shows characteristics of α-type pheromone precursors. Our results indicate that this unusual a-type peptide pheromone precursor is indeed essential for male fertility upon mating and thus pheromone response although its genomic locus indicates that after loss of the original a-type pheromone precursor this protein might have assumed the respective function. Based on its characteristics and the finding of similar proteins in heterothallic *Fusarium* spp., we propose a new class of peptide pheromone precursors denominated h-type (hybrid) pheromone precursors.

## Results

### HPP1 encodes a 5.1 kDa peptide pheromone precursor

**Fig. 1:**
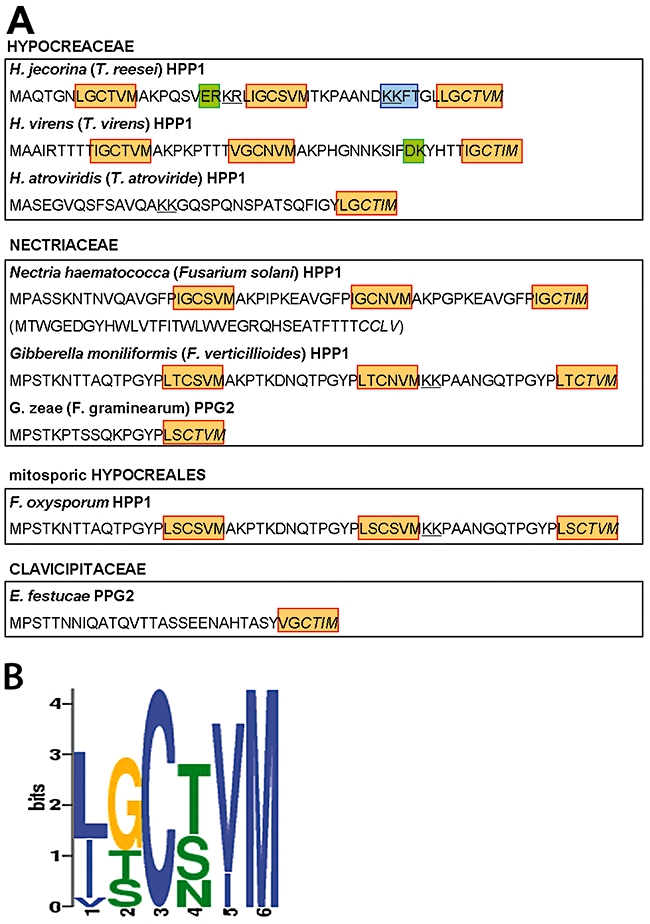
Sequence characteristics of HPP1 and its presumed functional homologues.A. Primary structure of the presumed h-type (HPP1 orthologues) and a-type peptide pheromone precursor proteins in *Hypocrea jecorina* (*Trichoderma reesei*), *Hypocrea virens* (*Trichoderma virens*), *Hypocrea atroviridis* (*Trichoderma atroviride*), *Nectria haematococca* (*Fusarium solani*), *Gibberella moniliformis* (*Fusarium verticillioides*), *Fusarium oxysporum*, *Gibberella zeae* (*Fusarium graminearum*) and *Epichloe festucae*. Genomic coordinates of these proteins are given in Table S1. The repeated motif is shaded in red, putative Kex2-peptidase sites are underlined. The characteristic C-terminal CAAX domain is given in italics. Acid-basic pairs are shaded in green. The cAMP- and cGMP-dependent phosphorylation site of *H. jecorina* HPP1 is shaded in blue.B. Consensus sequence (*E*-value 3E-32) as determined from the h-type peptide pheromone precursors given above by MEME. Hydrophobic amino acids are given in blue, polar non-charged amino acids are given in green and glycine (also polar and non-charged) is given in orange. [(Bailey and Elkan, 1994); http://meme.sdsc.edu]. Characteristically, h-type peptide pheromone precursors comprise three copies of the consensus sequence, one of them at the extreme C-terminus, thus creating a CAAX domain.

The gene encoding HPP1 was originally isolated as an EcoRII–cDNA fragment, *cfa11C* (GenBank Accession No. CF653649), reported to be associated to cellulase signal transduction in *H. jecorina* (Schmoll *et al*., 2004). Transcription start point and 3′ UTR were mapped by 5′ and 3′ RACE resulting in a complete cDNA of the respective gene. An upstream in frame stop codon was detected, indicating that RACE amplified the full-length cDNA (Kozak, 1996). The 591 bp full-length cDNA contains a 5′ UTR of 82 nt, an ORF of 144 nt and a 3′ UTR of 347 nt. This unexpectedly long 3′ UTR is not without precedent as such long 3′ UTRs have also been found in a-type pheromone precursor genes of other organisms, for example, with *mfa-1* of *N. crassa* (Kim *et al*., 2002), *mf2-1* of *C. parasitica* (Zhang *et al*., 1998), MF1-1 of *M. grisea* (Shen *et al*., 1999) or *ppg2* of *S. macrospora* (Poggeler, 2000). A polyadenylation signal is lacking. The first ORF starts with an ideal Kozak consensus and encodes a 48 aa polypeptide (Fig. 1A) with a deduced Mr of 5.1 kDa and a predicted isoelectric point of 9.63. It belongs to the α/β-type of proteins as revealed by a Garnier/Robson blot (Garnier *et al*., 1978) with three predicted β-sheets and two helices (data not shown). Interestingly, the amino acid sequence of all three β-sheets is almost identical: (L/I)GC(S/T)VM. Near the terminus, it contains a consensus sequence for cAMP- and cGMP-dependent phosphorylation (T_40_), which has not been detected in other a-type pheromone precursors, and a CAAX motif (Glomset *et al*., 1990; Powers, 1991) for covalent attachment of a prenyl group. As this motif ends with a methionine, the polypeptide is likely to be farnesylated (Moores *et al*., 1991). In order to appreciate the particular structure of this unusual peptide pheromone, the gene was named hybrid peptide pheromone precursor gene 1 (*hpp1*) (GenBank Accession No. AAQ95218).

Interestingly, a second ORF occurs in the 3′ UTR (nts 385–468), which like the first ORF, starts with a good Kozak consensus (Kozak, 2002). According to the rule proposed by Kozak (Kozak, 1996) ‘not the best but the first start codon’ it is unlikely that this ORF is functionally translated. Alternatively, a hypothesis by Turina and coworkers (Turina *et al*., 2003) suggests that such additional ORFs may be important for asexual sporulation. However, deletion of the area spanning the full mRNA sequence of *hpp1* (see below) does not alter asexual sporulation in *H. jecorina* and hence renders this hypothesis unlikely for this fungus.

Both the size of the polypeptide as well as the C-terminal prenylation signal are consistent with a function of HPP1 as a peptide pheromone precursor. Within the promotor region of *hpp1* two pheromone response elements (5′-CAAAG-3′), were found at positions −639 and −610 on the template strand. These sequences were identified in the basidiomycete *U. maydis* and are frequently present in the vicinity of pheromone inducible genes. Both are necessary and sufficient for pheromone induction (Hartmann *et al*., 1996; Urban *et al*., 1996), but so far have not been shown to be functional in ascomycetes.

This region further comprises GATA-factor binding sites (5′-HGATAR-3′) at −716 and −501, a CCAAT-box at −370 and a TATA-box at −133 relative to the ATG. Blastn and tblastn searches of the *Trichoderma reesei* genome database v2.0 (http://genome.jgi-psf.org/Trire2/Trire2.home.html) confirmed that *hpp1* is present in the genome as a unique copy. Additionally, Southern blotting with a probe spanning the *hpp1* ORF confirmed this result (data not shown). SecretomeP 2.0 (http://www.cbs.dtu.dk/services/SecretomeP/) predicts no signal sequence, but suggests non-classical secretion of HPP1. Analyses of a-factor-like peptide pheromone precursors of the heterothallic ascomycetes *C. parasitica* and *M. grisea* revealed a conserved C-terminal XYCY/IVM region (Zhang *et al*., 1998; Shen *et al*., 1999), which corresponds well with the HPP1–C-terminus. However, the threefold repeated motif of HPP1 as well as the presence of two Kex2-protease sites (which were, however, only found in *H. jecorina* HPP1) also hints to characteristics of α-factor-like pheromone precursors, although the motif does not fit to the respective consensus motif of WCXXGXXCW (Zhang *et al*., 1998; Shen *et al*., 1999).

### HPP1 represents a novel type of peptide pheromone precursors

As the particular structure of HPP1 suggested that it could be the first representative of a so far unidentified type of peptide pheromone precursors, we were interested whether this type is also present in genomes of other fungi. Therefore, all fungal genome databases available at the BROAD Institute (http://www.broad.mit.edu/annotation/), the Joint Genome Initiative of the US Department of Energy (http://genome.jgi-psf.org/), the *Fusarium sporotrichioides* EST-database at the University of Oklahoma (http://www.genome.ou.edu/fsporo_blast.html) and the *Epichloe festucae* genome database at the University of Kentucky (http://www.endophyte.uky.edu) were screened for proteins with similar characteristics. Hence, the genomes screened cover a broad range of phyla such as basidiomycota, ascomycota, zygomycota and chytridiomycota. Orthologues of HPP1 have so far only been detected in fungi belonging to the order of Hypocreales (Fig. 1A). Analysis of these polypeptides by MEME [(Bailey and Elkan, 1994); http://meme.sdsc.edu] revealed a 6-amino-acid conserved motif with an *E*-value of 3E-32 (Fig. 1B). Interestingly, this consensus indicates a preference of the first aliphatic amino acid in the CAAX motif for a polar, uncharged one (threonine, serine or asparagine). Therefore, we propose the prenylation site in the MAT1-2 mating system in Hypocreales to be a CPAX motif (p for polar). In agreement with the proposal by Reiss *et al*. (1991), this CPAX motif may be processed by a farnesyl transferase with different specificity from those dealing with CAAX domains. Nevertheless, a longer stretch of higher sequence similarity between closely related species can be found in some cases. While *Hypocrea virens* (anamorph *Trichoderma virens*) has a close orthologue to HPP1, the putative HPP1 orthologue of *Hypocrea atroviridis* (anamorph *Trichoderma atroviride*) found at the syntenic locus only comprises one consensus motif and does not share the characteristics of HPP1. The availability of ESTs from both *H. virens* and *H. jecorina* confirms that the respective genes are expressed. Besides those closely related species, HPP1 orthologues are also present in the heterothallic *Gibberella moniliformis* (*Fusarium verticillioides*), *Nectria haematococca* MPVI (*Fusarium solani*) and the reportedly asexual *Fusarium oxysporum*, but not in the homothallic *G. zeae* (*Fusarium graminearum*) or the asexual *F. sporotrichioides*. The occurrence of genes encoding proteins with similar characteristics as HPP1 supports our proposal of the novel class of h-type peptide pheromone precursors (hybrid-type), which is characterized by the occurrence of three copies of the consensus motif, one of them located at the extreme C-terminus thereby creating a CAAX domain for protein prenylation.

Alignment and phylogenetic analysis of HPP1 with its newly identified orthologues and a-type peptide pheromone precursors from other organisms indicates that HPP1 represents a novel variant in the order Hypocreales. However, the low conservation of these proteins considerably decreases the reliability of this analysis (data not shown). Similarly, we also cannot rule out that small proteins with similar characteristics remain to be detected in other fungi, but escaped our screening. HPP1 shows no significant similarity to genes available in GenBank or present in other than the above-mentioned fungal genome databases.

### *Hypocrea jecorina* has a normal α-factor-like peptide pheromone precursor

**Fig. 2:**
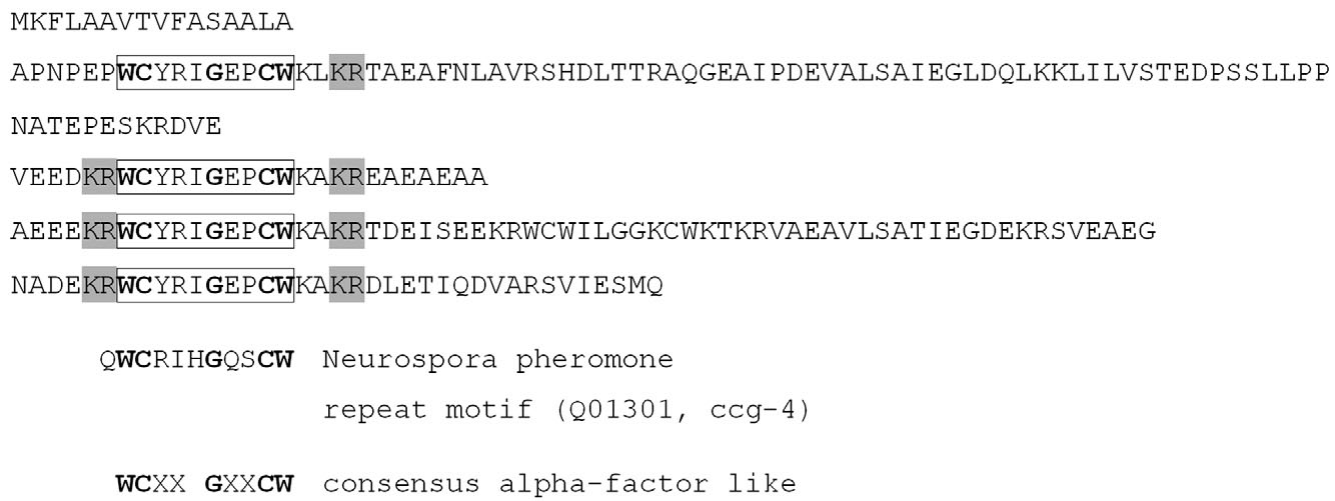
Primary structure and consensus sequence of the pheromone precursor protein PPG1. The repeated motif and thus predicted mature pheromone is boxed and the Kex2-peptidase recognition sites are shaded.

The finding of an unusual a-type pheromone precursor prompted us to analyse whether the second type of peptide pheromone precursors would match the common characteristics for α-type peptide pheromone precursors. A 286-amino-acid polypeptide with distant homology to other α-type pheromone precursors was found to be encoded by a gene located on scaffold 3 (tre9948; Protein ID: 104292) in the *Trichoderma reesei* genome database v2.0. An N-terminal signal sequence is predicted for this protein [SignalP; http://www.cbs.dtu.dk/services/SignalP/; (Emanuelsson *et al*., 2007)]. It comprises four copies of the 11 aa presumed mature pheromone WCYRIGEPCW (Fig. 2), which fits the consensus of WCXXGXXCW (Zhang *et al*., 1998; Shen *et al*., 1999). As in many α-type pheromone precursors, also Kex2-cleavage sites (Rockwell *et al*., 2002) are present in the respective ORF. The α-type pheromone precursors of *S. cerevisiae* are known substrates of the Kex2-protease (Fuller *et al*., 1988), which has been shown to be essential for normal mating by α-strains in *S. cerevisiae* (Leibowitz and Wickner, 1976). The promotor of this gene comprises three pheromone response elements (Hartmann *et al*., 1996; Urban *et al*., 1996) at −949, −733 and −459 relative to the ATG. Based on these characteristics, we denominated this putative α-type pheromone precursor gene as *ppg1* (peptide pheromone precursor gene 1).

No further gene corresponding to the characteristics of α-type pheromone precursors was found in the genome sequence of *H. jecorina*. We conclude that it is the only α-type pheromone precursor gene of *H. jecorina*.

### The locus of *hpp1* differs from those found in related species

**Fig. 3:**
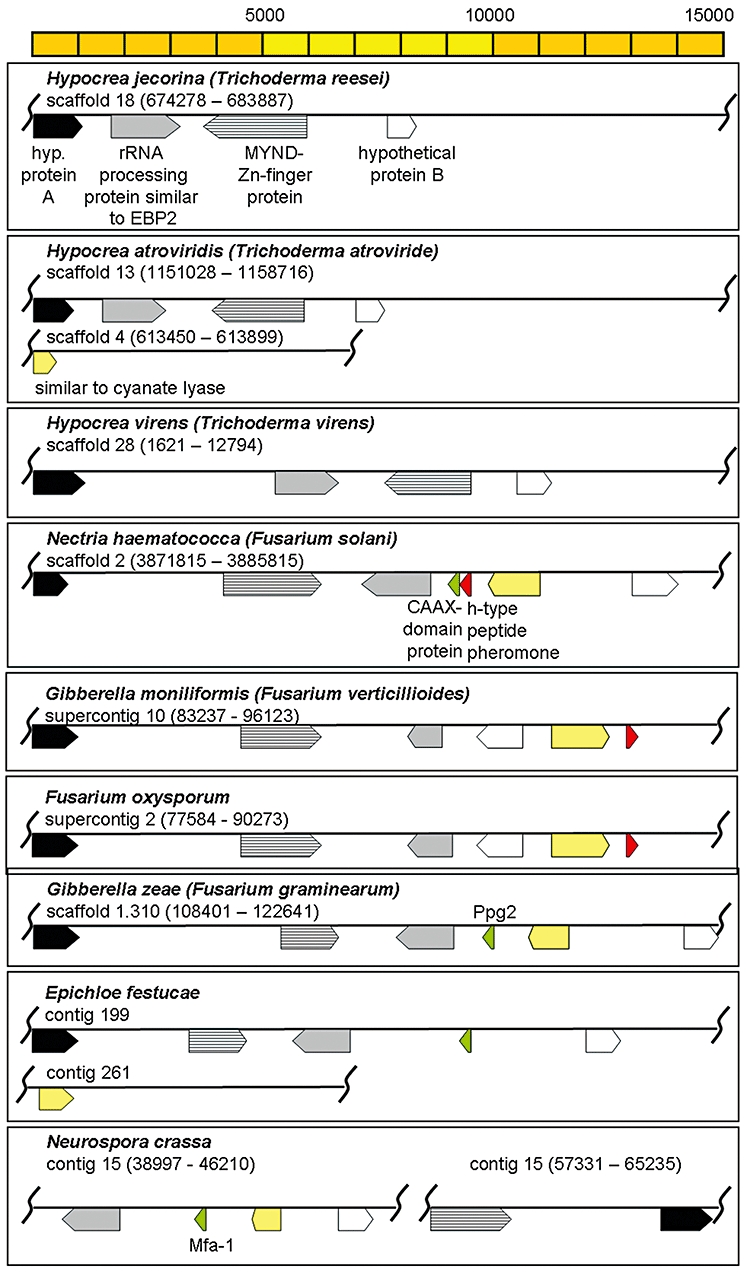
Schematic representation of the genomic locus of a-type peptide pheromone precursors. Genomic loci are given for *Nectria haematococca*, *Gibberella moniliformis*, *Fusarium oxysporum*, *Gibberella zeae*, *Epichloe festucae* and *Neurospora crassa* and compared with the respective locus in *Hypocrea jecorina*, *Hypocrea atroviridis* and *Hypocrea virens*. Homologous genes are represented in the same design. Predicted h-type peptide pheromone precursor genes are given in red, CAAX-domain protein encoding genes representing characterized or predicted a-type peptide pheromone precursor genes are given in green and (putative) cyanate lyase encoding genes are given in yellow. With the exception of *Neurospora crassa mfa-1* (GenBank Accession No. AAN03594) and *G. zeae ppg2* (Lee *et al*., 2008) the genes within these loci encode yet uncharacterized/predicted proteins. Therefore, instead of Accession No., the location within the respective scaffold of the genome is given. Precise genomic coordinates are available in Table S1.

As the gene encoding the respective a-factor pheromone precursor gene was found in the vicinity of a cyanate lyase gene in *N. crassa*, *M. grisea* and *G. zeae* (*F. graminearum*) (http://www.broad.mit.edu/annotation/fgi/), we expected the locus of *hpp1* to be similar to those fungi. We performed a detailed analysis and comparison of the respective area with the loci in *N. haematococca*, *G. moniliformis* (*F. verticillioides*), *F. oxysporum, G. zeae* (*F. graminearum*)*, E. festucae* and *N. crassa* including four flanking genes: one encoding a MYND-zinc finger protein (contains domain IPR002893), one homologue of the rRNA processing protein 2 (EBP2) and two hypothetical proteins designated A and B. This analysis revealed that neither a cyanate lyase homologue nor *hpp1* nor another CAAX-domain protein was present within this locus in *H. jecorina*, while the flanking genes (hypothetical proteins A and B) are syntenic with *N. haematococca*, *G. zeae* and *E. festucae* (Fig. 3; for genomic coordinates of the respective genes see Table S1). A similar genomic structure as in the *Fusarium* spp. was detected in *M. grisea* and *Chaetomium globosum*. Also in these genomes potential a-type pheromone precursors comprising CAAX domains were found downstream of the respective cyanate lyases (*C. globosum*: MPSTTTQTKVPQSTNFNGY; *M. grisea*: MSPSTKNIPAPVAGARAGPIHYCVIM; see also Table S1). In *P. anserina*, the arrangement is slightly altered, but the locus comprises the same genes as mentioned above with the peptide pheromone precursor downstream of cyanate lyase and the EBP2 and MYND-zinc finger protein homologues next to each other (Table S1). The arrangement of the genes in this area in *H. jecorina* suggests a genomic rearrangement event, which led to the loss of both the cyanate lyase homologue and the original a-factor peptide pheromone precursor gene in *H. jecorina* (Fig. 3). A similar arrangement of this locus was also detected in the genome sequences of *H. virens* and *H. atroviridis*. Interestingly, in the latter organism the cyanate lyase gene is indeed present, albeit at a different locus. Moreover we found that both orientation and order of the genes within this locus are variable (i.e. not strictly syntenic) in *Neurospora, Epichloe, Podospora* and *Fusarium* spp., thus contradicting simply unaltered inheritage of this locus from a common ancestor, although the orientation of the EBP2 and MYND-zinc finger protein homologues remains the same. However, it is currently not known at which point in evolution this divergence happened and which is the first common ancestor before this rearrangement.

Because of the assumed genome rearrangement or recombination event it cannot be excluded that an ORF encoding a small CAAX-domain protein might have been retained within the area upstream of the promotor of the EBP2 homologue. We therefore searched all six reading frames within 1.5 kb of this area, but did not find ORFs meeting the requirements for a putative a-factor pheromone precursor. The same analysis was done for *H. atroviridis, H. virens* and all other fungi included in Fig. 3. If a cyanate lyase was found in the respective organism also the genomic area downstream of this gene was screened. The area between the cyanate lyase orthologue and the EBP2 orthologue in *N. haematococca* comprises an ORF coding for a protein with intriguingly similar characteristics to HPP1, i.e. the repeated motif and the CAAX domain. Nevertheless, within the respective region also one additional small CAAX domain (Fig. 1A, in brackets) protein is encoded, which could also function as a peptide pheromone precursor, although the respective HPP1 orthologue better fits the a-type consensus. Besides this gene, we did not detect a further pheromone precursor candidate in this analysis in any fungus analysed.

### *hpp1* is located at an unusual locus in the genomes of *Hypocrea (Trichoderma)* spp

**Fig. 4:**
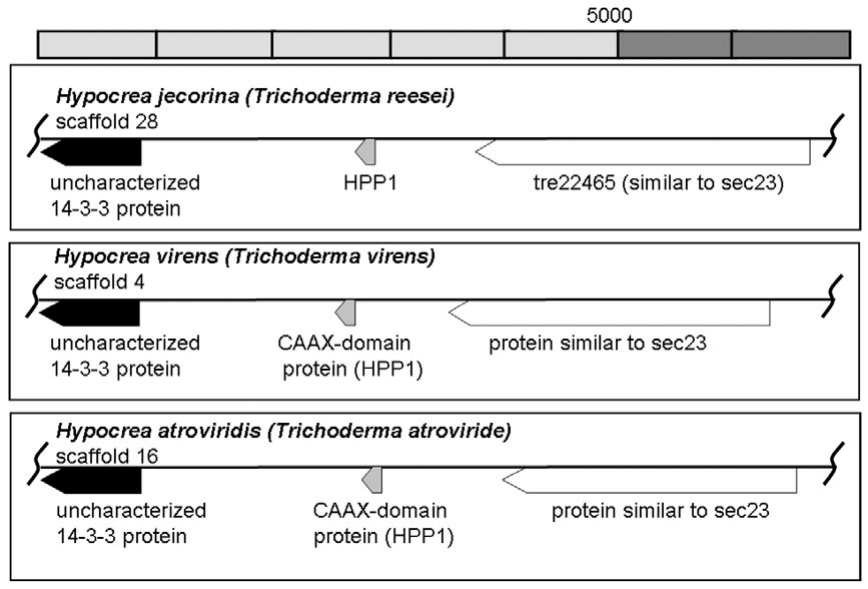
Schematic representation of the genomic locus of *hpp1* and its presumed functional homologues in *Hypocrea jecorina, Hypocrea atroviridis* and *Hypocrea virens*.

Interestingly, the loci containing the *Hypocrea* h-type peptide pheromone precursors are not syntenic with *Neurospora*, *Epichloe* or *Fusarium* spp. Within the *H. jecorina* genome, *hpp1* is located on scaffold 18 between tre22465, a putative homologue of *S. cerevisiae sec23* possibly involved in ER-Golgi trafficking and a yet uncharacterized 14-3-3 domain protein probably involved in intracellular signalling (Fig. 4). Homologues of the genes flanking *hpp1* have been found in *H. virens* and also a small ORF encoding a CAAX-domain protein with certain similarity to HPP1 (44% identity) was detected. The same analysis was done with the genome of the more distantly related *H. atroviridis*, but despite an expected nt-sequence similarity of approximately 80% to *H. jecorina* no ORF with significant similarity to *hpp1* was found in this fungus. Nevertheless the area between the respective *sec23* and the 14-3-3 orthologue revealed a CAAX-domain protein of comparable size with HPP1 and also at approximately the same distance to its flanking genes, but as expected without significant similarity to HPP1 (Figs 1A and 4). This protein contains only one h-type consensus motif and a recognition sequence for the Kex2-protease. Moreover, ESTs available on the *H. atroviride* genome homepage do not unequivocally support the functional transcription of this gene, because they would suggest transcription of another gene on the opposite strand. Therefore, devoid of further experimental support, assignment of this protein to h-type peptide pheromone precursors must be considered preliminary. Further analyses are necessary in order to elucidate whether these CAAX-domain proteins also assumed the function of the a-type peptide pheromone precursor in *H. virens* and *H. atroviridis*. In order to clarify, whether in *N. crassa, E. festucae* and *Fusarium* spp. the area around the genes homologous to those flanking *hpp1* could also be a potential site of (presumably) dormant CAAX-domain protein encoding genes, we searched the respective regions and found appropriate ORFs in *N. crassa* (sequence: MFIIRIRRAYRVASSRLERRIVGCVLT; ORF found in reverse orientation within the second intron of the ORF of NCU01317, a gene encoding a 60S ribosomal protein L12) downstream of the *sec23* homologue, as well as two such ORFs in the vicinity of the *sec23* homologue (sequence: MPPLLMPLKLCWID; upstream) and the 14-3-3 homologue (sequence: MVASRAQSCALK; downstream) in *G. zeae* (*F. graminearum*). Those genes however, did not comprise the triple motif of HPP1. Moreover the ORFs found in *G. zeae* are upstream (*sec23*) or downstream (14-3-3) of the respective gene, which – considering the locus in *Hypocrea* spp. – does not fit the order of genes in the *hpp1* locus. No ORFs for CAAX-domain proteins were found in these regions in *E. festucae* and *Aspergillus nidulans*.

Hence, the different loci yet similar characteristics of the h-type peptide pheromone precursors in *Hypocrea* spp. and *Fusarium* spp. rule out that the presence and functionality (see below) of HPP1 in *H. jecorina* represents an evolutionary artefact due to the deletion in the original a-type locus in *Hypocrea* spp.

### HPP1 is essential for male fertility

Although female sterility of the *T. reesei/H. jecorina* nature isolate QM6a causes certain inconveniences with the application of sexual development for strain improvement, the female sterile background of QM6a and all its progeny (including the frequently used QM9414) allows for investigation of the influence of putative regulators or conditions on male fertility. In case of a negative effect of a deletion on male fertility we would observe complete sterility of the respective strain. Thus, by comparison with the effect in the female fertile CBS999.97, it is possible to unequivocally distinguish between influences on male and/or female fertility.

**Fig. 5:**
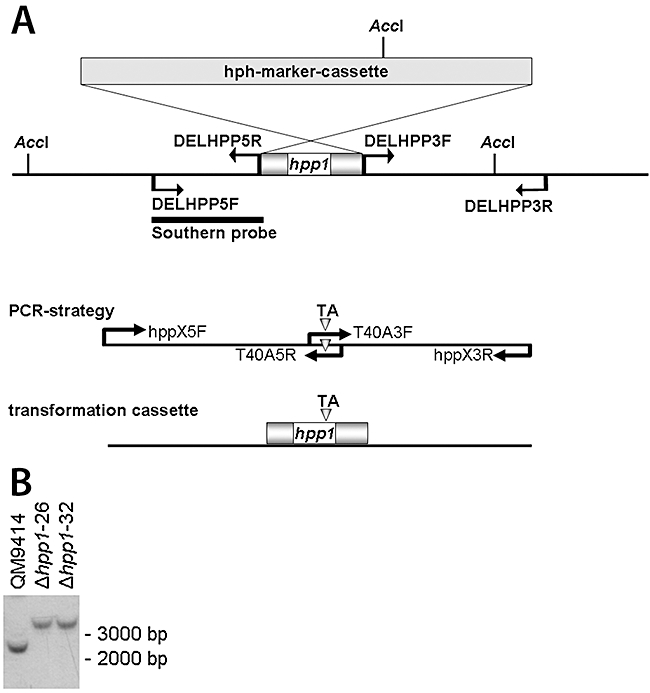
Construction of strains for analysis of the function of HPP1. A. Deletion of *hpp1* was achieved by replacing the sequence spanning the mRNA of *hpp1* by the *hph*-marker cassette. Successful deletion was evaluated by Southern blotting using AccI. The expected fragment size for the wild type is 2480 bp and for the deletion strains 3523 bp. A PCR fragment amplified with primers DELHPP5F and DELHPP5R was used a probe. Presence of the expected fragments and no additional bands indicates deletion of *hpp1* and shows no ectopically integrated cassettes. A PCR fragment amplified with primers DELHPP5F and DELHPP3R was used for retransformation of the deletion strain Δ*hpp1*. B. PCR-strategy for construction of a strain expressing HPP1 with an unfunctional cAMP-dependent phosphorylation site (*hpp1*T40A).

For a functional characterization of *hpp1*, we deleted the genomic region spanning the whole cDNA of *hpp1* in the female sterile QM9414 (MAT1-2) (Fig. 5) and retransformed the respective deletion mutant with a wild-type copy. No significant effects on growth or conidiation could be detected in any of these strains.

We screened for differences in the behaviour of *H. jecorina* CBS999.97 MAT1-1 upon confrontation with the female sterile strain QM9414 (MAT1-2) containing wild-type *hpp1* or the mutant strain deleted for the putative a-type peptide pheromone gene *hpp1* respectively, as described by Seidl *et al*. (Seidl *et al*., 2009). Additionally, we used a strain in which the cAMP-dependent phosphorylation site of HPP1 was rendered unfunctional (hpp1T40A), in order to test whether phosphorylation of HPP1 is relevant for its function in sexual development. Replacement of the threonine within the putative cAMP-dependent phosphorylation site of HPP1 by alanine prevents phosphorylation and hence renders this phosphorylation site unfunctional.

**Fig. 6:**
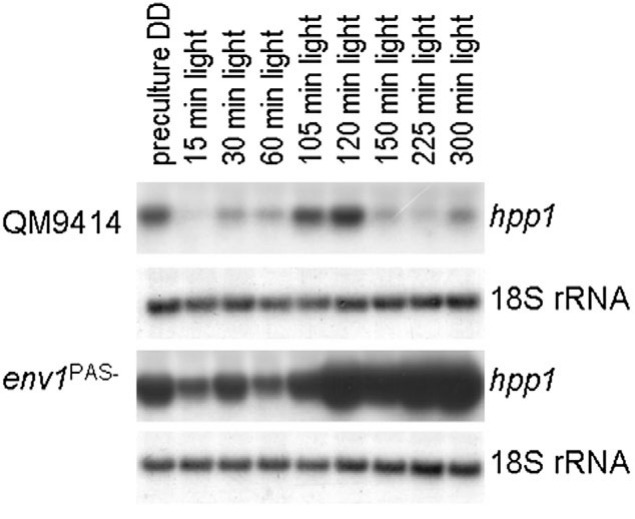
Transcription of *hpp1* in wild type and an ENV1 mutant strain *env1*^PAS−^. Strains were grown for 24 h in constant darkness and mycelia were harvested after illumination for the time given in the figure. For Northern blots, 20 µg of RNA were loaded per lane, and hybridizations were performed with PCR fragments spanning the ORF of *hpp1*. Hybridization with an 18S rRNA probe is shown as a control.

As analysis of transcription of *hpp1* in the strain *env1*^PAS−^ (Schmoll *et al*., 2005), which lacks the PAS domain of the light regulatory protein ENVOY, revealed a strong upregulation of *hpp1*-transcription upon cultivation in light (Fig. 6) we also added this strain to our analysis.

**Fig. 7:**
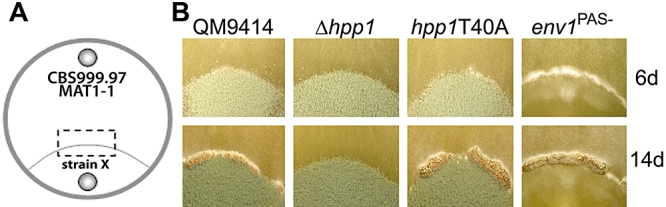
Fruiting body formation in mutants of *hpp1*. A. Schematic representation of the experimental setup for induction of sexual development between CBS999.97 (MAT1-1) and MAT1-2 strains derived from QM9414 to be tested for fruiting body formation. B. Perithecia formation on the border of confrontation between *H. jecorina* CBS999.97 (MAT1-1), the wild-type strain *H. jecorina* QM9414 (MAT1-2), Δ*hpp1* (MAT1-2), *hpp1*T40A (MAT1-2) and the mutant deleted for the PAS domain of the light regulatory protein ENV1, *env1*^PAS−^ (MAT1-2). Strains were grown on malt extract agar for the time indicated. The plate assays were done at least in triplicates; one representative plate is shown in the figure.

In this test we observed that – in accordance with our earlier study (Seidl *et al*., 2009) – fruiting body formation on the border of confrontation with *H. jecorina* CBS999.97 started after day 5 for QM9414. Figure 7 shows the beginning of fruiting body formation on day 6 and mature fruiting bodies on day 14 after inoculation. The formation of stromata did not occur with the *hpp1* deletion strain Δ*hpp1*, whereas it was enhanced and started earlier (as can be observed on day 6) as compared with the wild-type strain QM9414, with the strain *env1*^PAS−^ that overexpresses *hpp1* (see also below). However, we presently cannot be sure whether this is a direct or indirect effect of *env1*, because this gene has numerous regulatory targets (Schuster *et al*., 2007). Fruiting body development and ascospore discharge were indistinguishable from wild type in hpp1T40A and we therefore conclude that the cAMP-dependent phosphorylation site of HPP1 is not relevant for sexual development.

In order to obtain information on the potential processing of HPP1, we tested whether the two KEX2-protease processing sites might be essential for the function of HPP1. As loss of the positive charge at amino acid position P1 results in a drastic drop in catalytic efficiency at this site (Rockwell *et al*., 1997; 2002;), we exchanged the P1 residue in the respective site for asparagine (uncharged) in each site individually, resulting in strains hpp1R22N and hpp1K38N and in both sites, resulting in strain hpp1DX. We used two to six independent transformants for every mutation to check for fertility by crossing with CBS999.97 MAT1-1. Crosses were repeated twice. Despite lack of one or both KEX2-processing sites, none of the mutations abolished fruiting body formation or ascospore discharge (Fig. S1). Hence, we conclude that either the KEX2-processing sites in HPP1 are not essential for processing or the unprocessed pheromone precursor is still recognized by the mating partner.

Wild-type behaviour was completely restored in an *hpp1* retransformant. Thus, we conclude that HPP1 represents the only functional a-type peptide pheromone precursor of *H. jecorina* that obviously has assumed a-type function and is essential for male fertility of QM9414. As neither fruiting body formation nor ascospore discharge was altered in a strain deleted for *ppg1* (Fig. S2), this gene – as expected – does not play a role in male fertility of QM9414 (MAT1-2).

### HPP1 does not generally interfere with sexual development

**Fig. 8:**
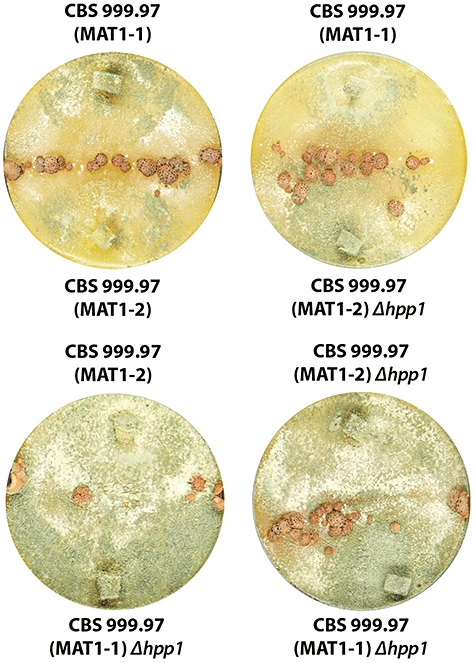
Effect of the *hpp1* deletion in *H. jecorina* CBS 999.97 on fruiting body formation. Deletion of *hpp1* in *H. jecorina* CBS999.97 (MAT1-2) does not significantly alter fruiting body formation upon mating with CBS999.97 (MAT1-1). Also deletion in the CBS999.97 (MAT1-1) strain or in both mating partners did not lead to complete sterility of either strain. Strains were grown for a period of 14 days on malt extract agar plates at 25°C in daylight.

As deletion of *hpp1* abolished mating of QM9414 (MAT1-2) with *H. jecorina* CBS999.97 MAT1-1, the question arose, whether it could have a more general function in sexual development and would possibly be essential for this process. Because of the female sterility of QM9414 [which is a derivative of the female sterile QM6a described in (Seidl *et al*., 2009)] we could not distinguish, if *hpp1* would only be essential for male fertility (as would be expected for an a-type peptide pheromone) or if it would have a more general function in sexual development. Therefore, we constructed deletion strains of *hpp1* in a female fertile background (*H. jecorina* CBS999.97 MAT1-1 and MAT1-2) and analysed the influence of lack of *hpp1* on sexual development in these strains. In both cases, we found that sexual development (fruiting body formation and ascospore discharge) is not perturbed (Fig. 8) and hence we conclude that *hpp1* is essential for male fertility, but not sexual development in general. This result further confirms that despite its unusual structure, *hpp1* has assumed the function of an a-type peptide pheromone in *H. jecorina*.

### *hpp1* and *ppg1* are transcribed during sexual development

**Fig. 9:**
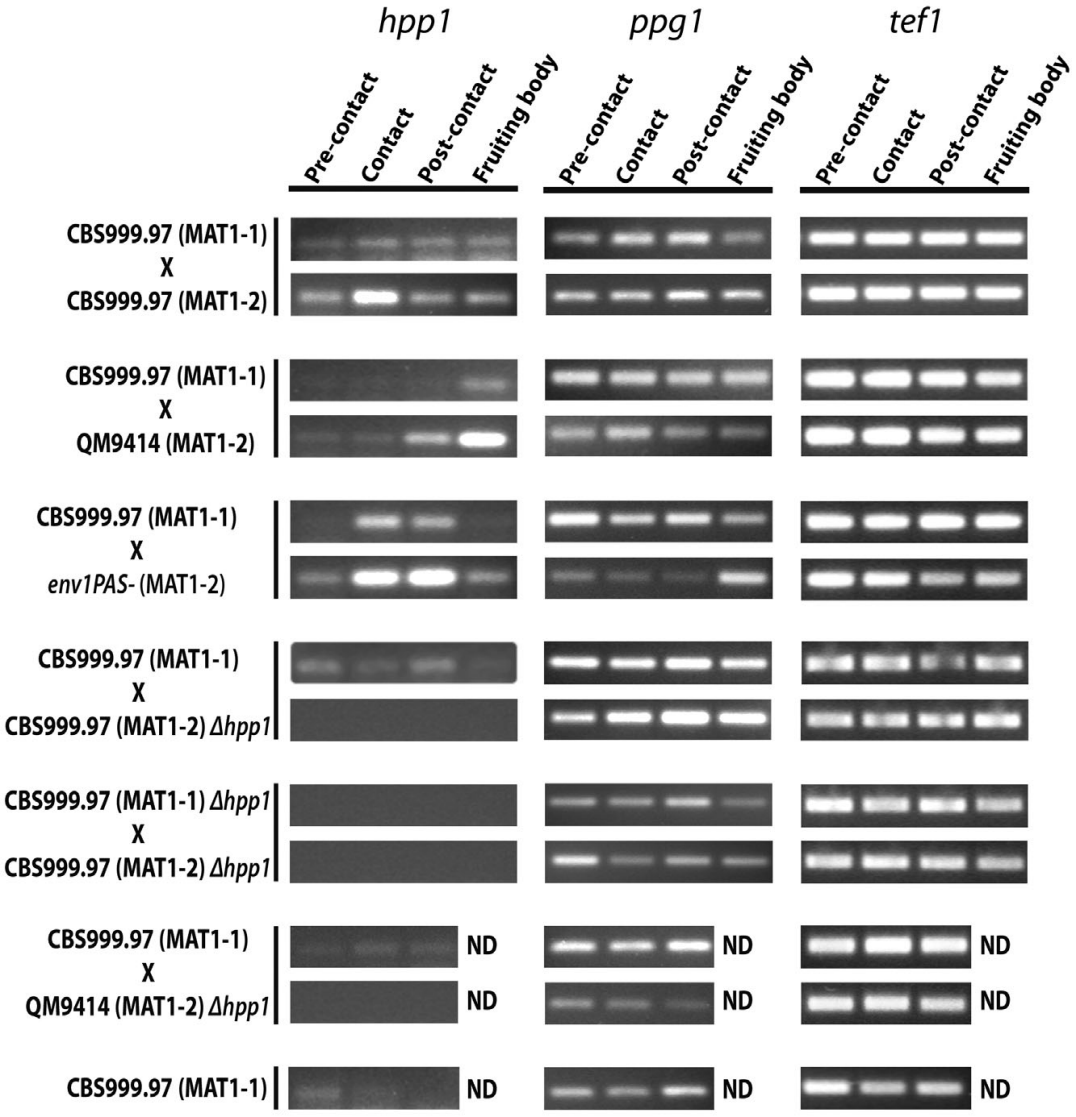
Transcription of *hpp1* and the α-type peptide pheromone precursor gene *ppg1* during development in QM9414 (MAT1-2), Δ*hpp1* (MAT1-2), *env1*^PAS−^ (MAT1-2), deletion mutants of *hpp1* in CBS999.97 (MAT1-1 or MAT1-2) and wild-type CBS999.97 (MAT1-1 or MAT1-2). Total RNA was isolated from these strains before contact (pre-contact), upon contact with the mating partner (contact), after contact (post contact) and after fruiting body formation (fruiting body). For the control CBS999.97 MAT1-1, which was grown in the absence of a mating partner, and upon confrontation of CBS999.97 (MAT1-1) with Δ*hpp1* the transcription of *hpp1* and *ppg1* after fruiting body formation (which did not occur) was not determined (ND). Total RNA (containing residual DNA) was used as positive control and DNase I treated non-reverse transcribed RNA as negative control for RT-PCR. Results for *tef1* (translation elongation factor 1-α) are shown as controls.

For the heterothallic ascomycetes *N. crassa* (Bobrowicz *et al*., 2002), *M. grisea* (Shen *et al*., 1999) and *C. parasitica* (Zhang *et al*., 1998), it was shown that the peptide pheromone precursors are expressed in a mating-type-specific manner. In order to test whether this is also the case for the heterothallic *H. jecorina*, we analysed transcription of both peptide pheromone precursors during development in QM9414 (MAT1-2), QM9414 Δ*hpp1* (MAT1-2), CBS999.97 MAT1-1 and deletion mutants of *hpp1* in CBS999.97 MAT1-1 and MAT1-2 in daylight before contact (day 3), at contact (day 4), after contact (day 5/6) and after fruiting body formation (day 7/8) (Fig. 9).

Interestingly, we found that both *hpp1* and *ppg1* are transcribed during development of *H. jecorina* and that this transcription is not dependent on the mating type, neither for *hpp1* nor for *ppg1*. Nevertheless, transcription of *hpp1* appears to be slightly enhanced in MAT1-2 strains. In support of the enhanced fruiting body formation upon confrontation of *env1*^PAS−^ (MAT1-2) with CBS999.97 MAT1-1 as described above, transcription of *hpp1* is enhanced in *env1*^PAS−^ as compared with CBS999.97 MAT1-1 under these conditions. Presence or absence of *hpp1* in the genome of the mating partner only had a slight, if any influence on transcription of *hpp1* or *ppg1* in the other partner.

Interestingly, we found pheromone response elements both in the promotors of *hpp1* and *ppg1* (see above), thereby offering a possible explanation for the transcription of *ppg1* in this strain, which would not be expected in a MAT1-2 strain. Nevertheless, it should be kept in mind that the function of pheromone response elements has only been shown in the basidiomycete *U. maydis* (Hartmann *et al*., 1996; Urban *et al*., 1996) and is not necessarily comparable in ascomycetes.

## Discussion

Sexual development represents one of the most important accomplishments in evolution. It is predominantly meant to improve the chances of survival of a species in a changing environment, because recombination through meiosis enables enlarging the gene pool and hence acquiring features crucial for adaptation to the altered conditions. Thus, sexual development is a significant evolutionary benefit of a given organism and specifically in fungi. In contrast, numerous examples of obviously only asexually propagating fungi are known. Although this might be due to inappropriate experimental conditions in screening assays, it is equally possible that sexual development indeed does not occur or is only a very rare event with these organisms and thus of low relevance for survival. Also, the existence of truly asexual organisms is being challenged because of the lack of conclusive methods to prove asexuality (Taylor *et al*., 1999). *T. reesei* was initially only known from a single isolate (QM6a) and all strains currently in use in research and industry are derived from this single isolate. Despite the elucidation of the teleomorph of *T. reesei* more than a decade ago (Kuhls *et al*., 1996), sexual development of the fungus has only recently received closer attention. In this respect the case of *H. jecorina* (*T. reesei*) QM6a is intriguing, as this wild-type isolate is female sterile (Seidl *et al*., 2009) and appears to have lost its original a-type peptide pheromone precursor, the latter phenomenon being a common characteristic in the genus *Hypocrea*.

HPP1 was initially identified in a study towards elucidation of the signalling process related to cellulase induction (Schmoll *et al*., 2004). It was found to be differentially regulated between a cellulase producing and a non-producing strain. This raises the question as to whether *hpp1* may – besides acting as a pheromone – also be involved in the regulation of expression of cellulase genes. Although some physiological functions beyond mating have been suggested for peptide pheromone precursors (Spellig *et al*., 1994; Willer *et al*., 1995; Chikashige *et al*., 1997; Debuchy, 1999), an involvement in regulation of biosynthesis of extracellular enzymes has not been reported so far. Hence, it cannot be excluded that initiation of fertilization and availability of nutrients might be connected by some regulatory pathways.

Divergence between related species has been suggested to proceed by inversions of small genomic regions, which is supported by studies on yeasts (Seoighe *et al*., 2000; Huynen *et al*., 2001) as well as by comparison of the genomes of *P. anserina* and *N. crassa* (Silar *et al*., 2003). These genomic rearrangements, which affect the architecture of a genome and represent important evolutionary events are suggested to primarily occur at the replication forks and cause inversions, translocations and deletions (Andersson, 2000; Tillier and Collins, 2000). The fact that an essentially similar locus and also a similar rearrangement of genes in the expected a-type pheromone locus in *H. jecorina* was found both in *H. virens* and *H. atroviridis*, eliminates the possibility that the loss of an original a-type peptide pheromone precursor gene occurred during strain improvement of the parent strain *H. jecorina* QM6a to QM9414, the strain used in this study. This locus, as well as the potential translocation of *hpp1*, seems to be unique to *Hypocrea*; hence, these characteristics rather represent an early evolutionary development in this genus. As such translocation sites are often flanked by inversions or losses and gains of single genes (Tillier and Collins, 2000), this would also be a consistent explanation for the obvious deletion of the cyanate lyase and potentially the CAAX-domain protein in its vicinity observed in the locus syntenic to the a-type peptide pheromone precursor gene locus in other fungi. However, given the altered structure of HPP1 we cannot be sure whether the original a-type peptide pheromone precursor of *H. jecorina* was lost during this rearrangement and HPP1 has assumed its function or if a translocation event only led to the locus different from its orthologues (with similar characteristics) in other fungi. Interestingly, despite the synteny of the flanking genes (the sec23 orthologue and the 14-3-3 protein encoding gene) in *Hypocrea* species, the genomic area around the locus of *hpp1* seems to be quite variable at the nucleotide level, hence potentially enhancing the risk to loose this mating factor. Nevertheless, we could not find indications that this locus would be a hotspot for recombination, which could be concluded from this finding. Although the locus of *H. jecorina hpp1* is not syntenic in other fungi of the order Hypocreales, no indications for the involvement of a transposon have been detected. The genome of *H. jecorina* indeed contains traces of different types of transposons, but no active transposon was found (Martinez *et al*., 2008) and none of the candidates is located in proximity to the *hpp1* locus.

Pheromones are conserved proteins because of their similarity in other homothallic and heterothallic fungi (Poggeler, 2000). A comparison of the a-type peptide pheromone precursors from several organisms with the here proposed h-type peptide pheromones shows that the consensus for the a- and h-type pheromone precursors (so far only detected in closely related species) is rather loose, which contrasts with the high conservation of the α-type pheromone. It therefore seems possible that further h-type peptide pheromone precursors comprising three similar motifs, one of them constituting a CAAX domain at the C-terminus exist in other fungi, but have escaped detection so far because their repeated motif does not exactly match the consensus sequence developed in this study. At the present stage, we cannot rule out that the h-type peptide pheromone precursors described here represent a special variety of a-type peptide pheromone precursors, as they seem to have replaced the typical a-type gene in most cases. Moreover, investigation of maturation of these pheromone precursors will reveal whether their processing rather resembles a- or α-type pheromone precursors. Nevertheless, their distinctive structure stands out and necessitates unequivocal differentiation from known peptide pheromone precursors, even if they would turn out to be closely related to the known proteins.

It has been suggested for a-factor export that recognition by the responsible yeast transporter (Ste6p) may be solely dependent on the prenyl- and methyl-CAAX modifications, which would thus tolerate considerable variability in the structure of the a-type pheromones, although also a certain influence of the sequence has been reported (Huyer *et al*., 2006). This hypothesis was also supported by the finding that *S. cerevisiae* could process and functionally express the *Coprinopsis cinerea* a-factor pheromone precursor (Olesnicky *et al*., 1999). Therefore, processing of the HPP1-precursor protein resulting in a prenylated CAAX domain is likely to result in the functional pheromone.

While efforts to elucidate the nature of the natural mature and functional HPP1 are currently in progress, determination of the mechanism of HPP1 processing and export will still require future studies, because several possibilities for this process could be deduced from literature: It could be assumed that due to the detected KEX2-peptidase sites, intrinsically characteristic for α-type pheromone precursors, the maturation process of HPP1 resembles that of α-type peptide pheromones. If so, the mature pheromone would not comprise the C-terminus including the CAAX domain, which is essential for a-type peptide pheromone precursors and thus for the observed function of HPP1. Moreover, this processing would not result in similar peptides characteristic for α-type pheromones. Consequently our results, that the KEX2-peptidase sites are not essential for the function of HPP1 are in agreement with the hypothesis of an alternative maturation pathway which, however, does not rule out that these peptidase sites have functions other than in sexual development.

For *Coprinus cinereus* the presence of an acidic-basic pair was shown to be essential for pheromone function of the peptide, while C-terminal prenylation only increased the efficiency of the pheromone (Olesnicky *et al*., 1999). As neither the acid-basic pair nor the KEX2-protease sites have been detected in all proteins fulfilling the h-type characteristics, we consider it possible, that – alternative to the suggested processing sites – a maturation process comparable with that of rhodotorucine A in the basidiomycetous yeast *Rhodosporidium thoruloides* (Akada *et al*., 1989) and to the peptide pheromone precursors in *Tremella* spp. (Sakagami *et al*., 1981) may take place. The precursor protein of rhodotorucine A is a mixture of tandem pheromone sequences separated by CAAX-like spacers and represents an apparent union of the two separate forms of pheromone gene structure adopted by *S. cerevisiae* for the a- and α-factor genes. Processing of these precursors results in multiple mature peptide pheromones. Considering the characteristics of HPP1, a similar mechanism could also lead to the production of up to three prenylated peptides from the h-type pheromone precursor proteins. Analysis of the structures of the h-type peptide pheromones identified in this study (Fig. S3) showed that comparison with the structure and maturation of rhodotorucine A allows for a hypothesis on mature peptide pheromones in *Fusarium* spp. However, for *Hypocrea* spp. we can only speculate that the peptidase responsible for processing should act upstream of the consensus motif in a way that the mature peptide would comprise several amino acids additional to the consensus motif after prenylation. Still, in both cases, experimental information on mature peptide pheromones will be necessary to understand the respective maturation process. Unfortunately the enzymes responsible for the maturation of rhodotorucine A are not known and thus the presence and function of such a mechanism in species containing h-type pheromone precursor genes can at this time only be subject to speculation. Nevertheless, the structural similarities in peptide pheromone precursors between these basidiomycetes and species belonging to Hypocreales raise the question whether lateral transfer from basidiomycetes to an early ancestor of Hypocreales might be the reason for the presence of h-type peptide pheromone precursors within ascomycetes.

Interestingly, we found that both types of peptide pheromone precursors are transcribed during development in *H. jecorina*. In case of *hpp1*, this could be attributed to the unusual locus of the gene, which might have resulted in an altered expression pattern. However, although *hpp1*/HPP1 shows some unusual characteristics, our data provide substantial evidence that HPP1 has assumed the function of an a-type peptide pheromone precursor. Hence, its untypical expression pattern is unlikely to be due to HPP1 not being an a-type peptide pheromone precursor. Also the more or less constitutive transcription of *ppg1* in both QM9414 and CBS999.97 was rather unexpected. Nevertheless, *ppg1* seems not to interfere with the initiation of a mating response, which is in accordance with a study on post-transcriptional regulation of peptide pheromone expression in *P. anserina* (Coppin *et al*., 2005). Concordant with these findings, the result, that deletion of *hpp1* in a female sterile background abolishes perithecia formation upon contact with fertile strains as well as the phenotype of the *ppg1* deletion strain, rule out that PPG1 plays a role in the interaction of the MAT1-2 strain QM9414 with CBS999.97 (MAT1-1). As both peptide pheromone precursors are obviously not transcribed in a strictly mating-type-dependent manner, this can be considered rather a characteristic of *H. jecorina* than a hint as to a function other than pheromone precursors. It will be interesting to learn, how the presence of the mature HPP1 peptide as a mating signal is perceived, because it cannot unequivocally be assigned to either a- or α-type peptide pheromones and thus it can hardly be predicted which receptor is responsible for reception of this signal.

The lack of a common a-type peptide pheromone precursor gene in the *Hypocrea* spp. analysed in this study, initially suggested a fortunate recovery of the sex pheromone, which had been lost in a previous genomic rearrangement or recombination event. However, as proteins with similar characteristics as HPP1, also sharing the consensus sequence have been found in other fungi, which retained the genomic region presumably lost in *Hypocrea*, this hypothesis is highly unlikely. Most importantly, these genes are located within the syntenic genomic area, which comprises the a-type peptide pheromone precursor in closely related fungi, such as *N. crassa* and *M. grisea*. With the exception of *N. haematococca*, this locus did not comprise an additional, typical a-type peptide pheromone precursor, if an h-type was detected. Notably, the CAAX-domain protein of *N. haematococca* located downstream of the h-type protein, does not correspond well to the consensus sequence published for a-type peptide pheromone precursors (Zhang *et al*., 1998; Shen *et al*., 1999) and may thus be not functional.

Interestingly, all fungi containing an HPP1 orthologue are heterothallic, except *F. oxysporum*. However, this reportedly asexual fungus contains a set of MAT genes highly similar to those of the heterothallic *Gibberella fujikuroi* and unexpectedly these MAT genes are also expressed in *F. oxysporum* (Yun *et al*., 2000). Therefore, *F. oxysporum* may share characteristics of heterothallic fungi, although sexual development has not been reported so far. *G. zeae*, however, has a peptide pheromone precursor strikingly similar to the other *Fusarium* spp., but this protein comprises only one copy of the h-type consensus sequence. Therefore, it would not be considered an h-type peptide pheromone precursor, which would require three copies. Hence, we hypothesize that HPP1 could be a heterothallism-specific peptide pheromone precursor. Nevertheless, no protein with h-type characteristics has been detected in the heterothallic *E. festucae*, but again we found only one h-type consensus motif. Therefore, the occurrence of the h-type pheromones seems to be not a strict rule for heterothallic Hypocreales, but rather a peculiarity within this group. Consequently, only a detailed analysis of the genomes of several further homothallic and heterothallic species will reveal whether this hypothesis is admissible.

While the functionality of *H. jecorina* HPP1 as a peptide pheromone has been shown in this study, a similar function remains to be established for the other members of the novel group of h-type peptide pheromones. It will also be interesting to learn, whether proteins sharing these characteristics are present in other fungi and if their specific structure indicates functions beyond attracting a mating partner. Their size, and – besides the triple motif – low conservation as well as the lack of characterized domains in these small proteins may have prevented their discovery, as potential members of this class are unlikely to be found using standard tblastn searches. With the availability of further genomes of homothallic, heterothallic and asexual fungi it will also be possible to evaluate the specificity of h-type pheromones for heterothallic fungi. Further studies on these proteins and their functions will presumably reveal intriguing new insights into the mechanisms and determinants of mating in fungi.

## Experimental procedures

### Microbial strains and culture conditions

*Hypocrea jecorina* (*T. reesei*) wild-type strain QM9414 (ATCC 26921) as well as a strain lacking the PAS domain of ENV1 [*env1*^PAS−^; (Schmoll *et al*., 2005)] were used throughout this study. For the analysis of sexual development and mating response strain *H. jecorina* CBS999.97 (CBS, Utrecht, the Netherlands) was used. This strain (originally named G. J. S. 97-38) is phylogenetically close to *H. jecorina* QM6a, the predecessor of QM9414 and produces perithecia in axenic culture (Lieckfeldt *et al*., 2000). As the initial isolate deposited at CBS is a mixture of both mating types, purified and confirmed MAT1-1 or MAT1-2 strains derived from this isolate (Seidl *et al*., 2009) were used for this study. Strains were propagated on 3% (w/v) malt extract agar plates at 22°C in daylight to promote fruiting body formation. For transcript analysis, plates were covered with cellophane to facilitate harvesting of mycelia.

*Escherichia coli* JM109 (Yanisch-Perron *et al*., 1985) and *E. coli* ER1647 (Novagen, Madison, WI, USA) were used for DNA manipulations.

### Analysis of fruiting body formation

The strains were grown on malt extract agar. The test for initiation of fruiting body formation was done as described in (Seidl *et al*., 2009). Strains were inoculated on opposite sites of a malt extract agar plate and growth in daylight at 22°C was monitored for 14 days. All experiments were done at least in triplicates.

### Screening of a chromosomal library of *Hypocrea jecorina* QM9414

Isolation of chromosomal clones was performed according to the manufacturer's protocol (λBlueSTAR™ Vector System, Novagen). The *Trichoderma reesei* genome database (http://genome.jgi-psf.org/Trire2/Trire2.home.html) was used to evaluate the sequence.

### Nucleic acid isolation and blotting

Stains grown on malt extract agar plates in daylight at 22°C were harvested every 24 h as indicated in the legend to the figure. For DNA isolation, strains were grown in liquid malt extract medium, harvested by filtration and washed with tap water. Mycelia were snap frozen and ground in liquid nitrogen. DNA and RNA were isolated as described previously (Schmoll *et al*., 2004). Standard methods (Sambrook *et al*., 1989) were used for electrophoresis, blotting and hybridization of nucleic acids.

### Reverse transcriptase polymerase chain reaction

Five micrograms of total RNA was treated with DNase I (Fermentas, Vilnius, Lithuania), then the reaction was terminated by adding EDTA to a final concentration of 2.5 mM and incubation at 65°C for 10 min. The RevertAid™ First Strand cDNA Synthesis Kit (Fermentas) was used for first strand synthesis according to the manufacturer's protocol. Polymerase chain reaction (PCR) amplification was done with GoTaq Polymerase (Promega, Madison, WI, USA) and primers hppF and hppR spanning the reading frame of *hpp1* for 31 cycles, primers ppg1F and ppg1R for amplification of *ppg1* for 33 cycles and primers tefF and tefR for amplification of *tef1* for 25 cycles. For all primer sequences see Table 1. Total RNA (containing residual DNA) was used as positive control and DNase I treated RNA as negative control for PCR. Thirty-three PCR cycles were used for these controls. Individual samples were used for the positive control and yielded a fragment in the expected size. For the negative control pooled samples were used, along with a no template control, none of which showed an amplified fragment.

**Table 1:** Primers used throughout the study

Name	Sequence
hppF	5′-AATTCACAATGGCTCAAAC-3′
hppR	5′-GCTTGAAGCTGTTTACATG-3′
ppg1F	5′-TGGAGACGAAGGAGAAGACTG-3′
ppg1R	5′-GCGATGTGTGGTGATGGA-3′
tefF	5′-AGAAGGTCGGCTTCAACC-3′
tefR	5′-GGTAGTCGGTGAAAGCCTC-3′
DELHPP5F	5′-ATGGTACCTCAGAAGAGGTGGCCAAGAG-3′
DELHPP5R	5′-ACCTCGAGCTTGAGATTGAAGGCTGTTTG-3′
DELHPP3F	5′-AGAAGCTTATTCTATACATCATCATCTCGG-3′
DELHPP3R	5′-ATCTGCAGAGCTGGCAGGGTAACTTG-3′
matA2fw	5′-CTCGAGAGGGATATACACCAG-3′
matA2rv	5′-CTTCCTACACGGATGCCAGA-3′
mata1F	5′-GCGCACCACGGTATTTCATTG-3′
mata1R	5′-ATTTGCGCGGCTTGTATTGG-3′
hppT40A3F	5′-AGAAGTTC**G**CCGGCCTCCTG-3′
hppX3R	5′-GTCAGGTTGGCAAGAGACGG-3′
hppX5F	5′-CTGAACGAGCCCTGAGAGACGAC-3′
hppT40A5R	5′-CAGGAGGCCGG**C**GAACTTCTTG-3′
hppORF1F	5′-AATTCACAATGGCTCAAAC-3′
hppORF1R	5′-AGGCTTGAAGCTGTTTACATG-3′
HPAfull3F	5′-TCGAGAGGAAG**AAC**CTCATTGGCTGCAGCGTCATGACCAAGCCTGCCGCCAACGACAAG**AAC**TTCACCGGCCTCC-3′
HPAx3R	5′-CAACCTACCGACTGAACGAAG-3′
HPAx5F	5′-TTCCGCCCAGACCATCTTCAC-3′
HPAfull5R	5′-GGAGGCCGGTGAA**GTT**CTTGTCGTTGGCGGCAGGCTTGGTCATGACGCTGCAGCCAATGAG**GTT**CTTCCTCTCGA-3′
HPA1NF	5′-ATGTCGACGTGTCCTTGCAGACCTTC-3′
HPA1NR	5′-ATCGTACGCCTACCCACTGAACAAGAG-3′
HPAkr3F	5′-CGAGAGGAAG**AAC**CTCATTG-3′
HPAkr5R	5′-GCAGCCAATGAG**GTT**CTTCC-3′
HPAkk5R	5′-GGAGGCCGGTGAA**GTT**CTTGTC-3′
HPAKK3F	5′-CCAACGACAAG**AAC**TTCACCG-3′
HPP1RACE-A	5′-TGTTGTAAATGTGTACATGGTG-3′
HPPRACE-N	5′-TGTGTACATGGTGAATCCTG-3′
RASH18PB	5′-ACTATAGGGCGAATTGGG-3′
RACE-N	5′-GCGTAATACGACTCACTATAGGGCGAATTGGGTTTTTTTTTTTTTTTTT(AGC)-3′
HPPRACE3	5′-GTCGAGAGGAAGAGGCTC-3′

Restriction site introduced to facilitate vector construction are underlined; mutations are given bold and underlined.

### Preparation of *hpp1* deletion strains and retransformation

To obtain a deletion mutant of *hpp1*, QM9414 was transformed with plasmid pDELhpp1 in which the sequence spanning the complete mRNA of *hpp1* as determined by RACE is replaced by the *E. coli hph* gene under *H. jecorina* expression signals (Mach *et al*., 1994). This vector was constructed as follows: a 769 bp fragment of the 5′ flanking sequence of *hpp1* was amplified by PCR using primers DELHPP5F and DELHPP5R, the amplicons cleaved with Acc65I and XhoI (all restriction enzymes by Fermentas) and cloned into the Acc65I–XhoI sites of pBSXH (Schmoll *et al*., 2005), which contains the *pki1*p : *hph* : *cbh2*t cassette from pRLMex30 resulting in pDELhpp1H5. Thereafter, a 1275 bp fragment of the 3′ flanking region of *hpp1* was amplified by PCR using primers DELHPP3F and DELHPP3R and cloned into the HindIII–PstI sites of pBluescript SK+, the resulting plasmid cleaved with HindIII and SpeI, and the excised fragment thus obtained finally cloned into the respective sites of pDELhppH5 resulting in pDELhpp1. The plasmid was linearized by restriction digestion with SpeI and 10 µg were used for transformation (Gruber *et al*., 1990). Transformants were selected on plates containing 50 µg ml^−1^ hygromycin B (Calbiochem, Merck KGaA, Darmstadt, Germany). Fungal DNA was isolated from transformants using standard protocols and subjected to Southern analysis (using AccI) to verify replacement of the gene and the presence of no additional copies of the deletion cassette (Fig. 5). Two deletion strains (Δ*hpp1*-26 and Δ*hpp1*-32) were used and yielded similar results.

Complementation of Δ*hpp1* with the *hpp1* wild-type gene was achieved by cotransformation of a PCR fragment amplified with primers DELHPP5F and DELHPP3R comprising the entire gene and the *amdS* marker cassette (Penttila *et al*., 1987; Seiboth *et al*., 1997). Successful complementation was confirmed by PCR using primers hppF and hppR (see above). Wild-type behaviour was restored in the retransformants.

Deletion of *hpp1* in *H. jecorina* CBS999.97 (MAT1-2) was accomplished essentially as described above for *H. jecorina* QM9414. To obtain a deletion mutant of *hpp1* in *H. jecorina* CBS999.97 (MAT1-1) the single spore isolate of the primary deletion strain *H. jecorina* CBS999.97 (MAT1-2) Δ*hpp1* was crossed with the *H. jecorina* CBS999.97 (MAT1-1) wild-type strain. Progeny was isolated as described previously (Seidl *et al*., 2009). Hygromycine resistant single-ascospore clones were analysed by PCR to verify the deletion of *hpp1*. In order to determine the mating type of ascospore clones, primer pairs binding within the MAT1-1 or MAT1-2 mating-type loci were chosen. For the MAT1-1 mating-type locus the primers matA2fw and matA2rv and for the MAT1-2 locus the primer combination mata1F and mata1R was used.

### Construction of strains bearing an amino acid exchange in HPP1 and retransformation

For the mutated copy bearing a non-functional phosphorylation site a T→A amino acid exchange at position 40 was introduced using a PCR-based strategy. *Pfu*-polymerase (Fermentas) was used for amplification of the overlapping fragments. An 897 bp 3′ fragment was amplified using primers hppT40A3F and hppX3R, and a 1089 bp 5′ fragment using primers hppX5F and hppT40A5R. The fragments were purified using QIAquick PCR purification columns (QIAGEN, Hilden, Germany), combined and used as template for amplification of the complete 1966 bp mutated copy of *hpp1* with primers hppX5F and hppX3R. In this step, GoTaq Polymerase (Promega) was used to facilitate ligation.

All fragments were ligated into pGEM-T (Promega) and sequenced. The resulting plasmid (pHPPaaT40A) was linearized and used for cotransformation (Gruber *et al*., 1990) of Δ*hpp1*-32 with the amdS-marker gene cassette (Penttila *et al*., 1987; Seiboth *et al*., 1997). Integration of the mutated copy of *hpp1* into QM9414 Δ*hpp1* was analysed by PCR using hppORF1F and hppORF1R, which amplify a 167 bp fragment comprising the coding region of *hpp1*, along with DNA of QM9414 and Δ*hpp1*-32 as positive and negative controls respectively.

### Construction of strains lacking one or both KEX2-processing sites

The vectors for complementation of Δ*hpp1* with constructs for expression of altered versions of HPP1 lacking the first, the second or both KEX2-processing sites were constructed using a similar approach as described above.

For deletion of both KEX sites the PCR products amplified with primers HPAfull3F and HPAx3R or HPAx5F and HPAfull5R respectively, were purified, combined in equimolar amounts and used as template for a nested PCR using primers HPA1NF and HPA1NR. The resulting fragment was digested with BsiWI and SalI and ligated into Acc65I/XhoI digested pBSamdS, which contains an XbaI/SalI amdS fragment integrated into the corresponding sites of pBluescript SK+ (Stratagene).

Strategies for deletion of every individual KEX2 site were similar except that for deletion of the first KEX2 site (KR), primers HPAx5F and HPAkr5R or HPAkr3F and HPAx3R were used for primary PCR reactions. For deletion of the second KEX2 site (KK) primers HPAx5F and HPAkk5R or HPAkk3F and HPAx3R were used for primary PCR reactions. HPA1NF and HPA1NR were used for the secondary PCR in both cases and ligation into pBSamdS was done as described above.

All constructs were sequenced to confirm deletion of the KEX2 sites. Transformation cassettes were linearized using NotI and used for transformation into Δ*hpp1*-32 as described above.

### 5′ and 3′ RACE

For 5′ RACE, first strand synthesis was performed from 3 µg total RNA using the Reverse Transcription System (Promega) at 42°C for 1 h according to the manufacturers instructions with a gene-specific primer (HPP1RACE-A). Single-stranded RNA was digested using RNase A (Sigma-Aldrich, St Louis, MO, USA) and afterwards the reaction mixture was purified using the QIAquick PCR Cleanup Kit (QIAGEN). The DNA–RNA hybrid fragments were ligated into EcoRV digested pBLUESCRIPT (Stratagene, La Jolla, CA, USA) and PCR was performed with one nested gene-specific primer (HPPRACE-N) and RASH18PB, which binds to pBLUESCRIPT using Q-Taq and Q-Solution (QIAGEN). As a control chromosomal DNA was used to rule out, that contaminations of chromosomal DNA in the RNA-preparation result in false positives.

For 3′ RACE, first strand synthesis was performed using the Reverse Transcription System (Promega) according to the manufacturers protocol at 42°C for 1 h with primer RACE-N. The reaction mixture was cleaned up using the QIAquick PCR Cleanup Kit (QIAGEN). PCR and Nested PCR were performed according to standard protocols using a gene-specific primer (HPPRACE3). The resulting PCR fragments were ligated into pGEM-T (Promega) and sequenced.

### Bioinformatic analyses

Analysis of the genomic locus of *hpp1* and the loci of peptide pheromone precursors in related organisms was done by the aid of the genome databases published by the Joint Genome Institute of the US Department of Energy (http://genome.jgi-psf.org) and the Broad Institute (http://www.broad.mit.edu/annotation/fgi/). All fungal genome databases available on these sites including ascomycetes, basidiomycetes, zygomycetes and chytridiomycetes were analysed. Additionally, the *F. sporotrichioides* EST-database at the University of Oklahoma (http://www.genome.ou.edu/fsporo_blast.html) and the *Epichloe festucae* genome database at the University of Kentucky (http://www.endophyte.uky.edu) were used. Blast searches were done with the NCBI Blast server (http://www.ncbi.nlm.nih.gov/BLAST/). In any case, tblastn (*E*-value threshold set to 10.0; BLOSSOM62, PAM70 or PAM30 matrix) was applied in order to prevent a false negative result due to the small size and low conservation of the respective proteins, which may thus have escaped automatic annotation. Bidirectional blast was applied to confirm homology. Search for CAAX-domain proteins (Glomset *et al*., 1990) in all six reading frames was done with Generunner 3.00 (Hastings-Software Inc.) using the consensus sequence C{DENQ}[LIVM]X>.

For the phylogenetic analysis amino acid sequences were aligned with clustal X 1.81 (Thompson *et al*., 1997) and then visually adjusted using GeneDoc (Nicholas and Nicholas, 1997). Phylogenetic analyses were performed in MEGA 4 (Tamura *et al*., 2007) using the minimum evolution (ME) approach. The reliability of nodes was estimated by ME bootstrap percentages (Felsenstein, 1985) obtained after 500 pseudoreplications.
