# Supplementary material for: A novel class of peptide pheromone precursors in ascomycetous fungi
Source: Mol Microbiol. 2010 Jul 30;77(6):1483–501. doi: 10.1111/j.1365-2958.2010.07295.x (PMC3068285; doi:10.1111/j.1365-2958.2010.07295.x)
Supplement: Supplementary file 1 [file mmi0077-1483-SD1.pdf]

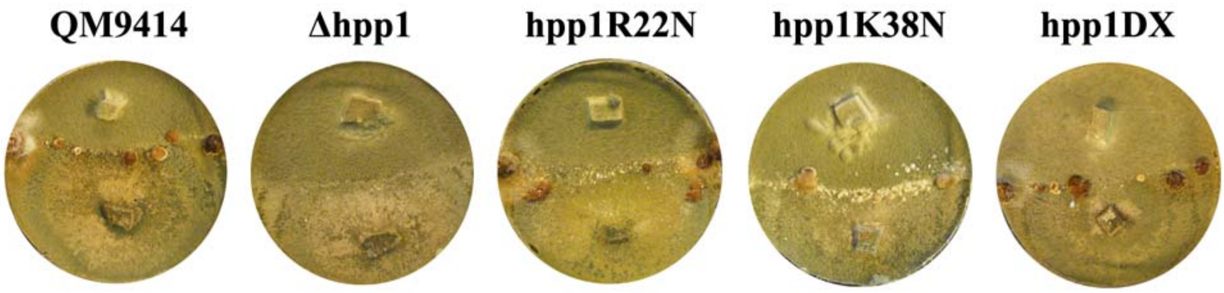

Figure S1

Deletion of the KEX2-peptidase sites does not abolish fruiting body formation. The *hpp1* deletion mutant Δhpp1 (shown here as negative control) was transformed with the different constructs resulting in strains expressing HPP1 lacking the first (hpp1R22N) or the second (hpp1K38N) or both (hpp1DX) KEX2-peptidase sites. Upon mating with the fertile strain CBS999.97 MAT1-1, fruiting body formation occurred with all strains except the negative control lacking *hpp1*.

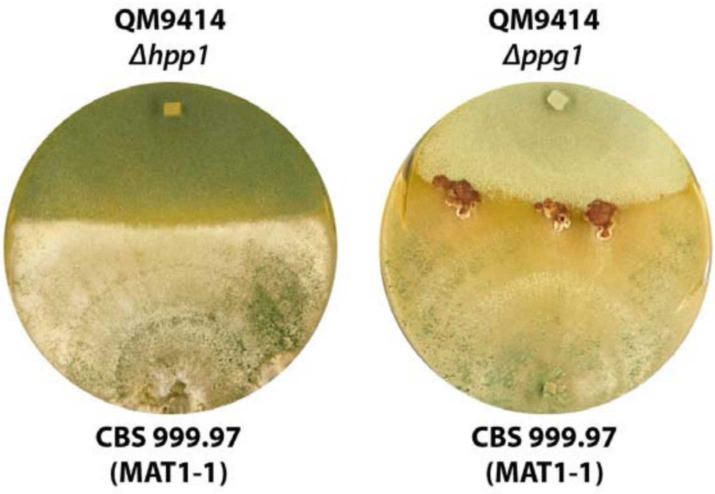

Figure S2

While deletion of *hpp1* in QM9414 abolishes fruiting body formation upon confrontation with CBS999.97, mating is still possible after deletion of the alpha type peptide pheromone precursor in QM9414 (MAT1-2) despite its female sterile background. Strain Δppg1 was obtained by A. Schuster (Vienna University of Technology).

A

***Rhodosporidium toruloides* Rhodotorucine A**

CAAX-domain like sequences

MVA **YPREISWTRNGC** TVAK **YPREISWTRNGC** TVSK **YPREISWTRNGC** TVSK **YPREISWTRNGC** TVA

leader sequence

mature prenylated peptide (YPREISWTRNGC)

TV(A/S)K ... spacer sequence

B

***H. jecorina* (*T. reesei*) HPP1**

```
      MAQTGNLGCTVM
  AKPQSVERKRLIGCSVM
TKPAANDKKFTGLLGCTVM
```

***H. virens* (*T. virens*) HPP1**

```
        MAAIRTTTTIGCTVM
         AKPKPTTTVGCNVM
AKPHGNNKSIFDKYHTTIGCTIM
```

***Nectria haematococca* (*Fusarium solani*) HPP1**

```
MPASSKNTNVQAVGFPIGCSVM
    AKPIPKEAVGFPIGCNVM
    AKPGPKEAVGFPIGCTIM
```

MPASSK **NTNVQAVGFPIGC** SVMAK **PIPKEAVGFPIGC** NVMAK **PGPKEAVGFPIGC** TIM

***Gibberella moniliformis* (*F. verticillioides*) HPP1**

```
MPSTKNT TAQTPGYPLTCSVM
   AKPTKDNQTPGYPLTCNVM
   KKPAANGQTPGYPLTCTVM
```

MPSTK **NTTAQTPGYPLTC** SVMAK **PTKDNQTPGYPLTC** NVMKK **PAANGQTPGYPLTC** TVM

***F. oxysporum* HPP1**

```
MPSTKNT TAQTPGYPLSCSVM
   AKPTKDNQTPGYPLSCSVM
   KKPAANGQTPGYPLSCTVM
```

MPSTK **NTTAQTPGYPLSC** SVMAK **PTKDNQTPGYPLSC** SVMKK **PAANGQTPGYPLSC** TVM

Figure S3

Rhodothorucine A-like processing of peptide pheromone precursors. (A) Structure of *Rhodosporidium toruloides* rhodothorucine A (Akada et al., 1989). CAAX domain like sequences are boxed and yellow, the mature peptide pheromone is given in bold and the spacer sequences in regular type. (B) Alignments of repeated h-type consensus and putative mature peptide pheromones (given in bold). Because of the lack of similarity of the partial sequences in *Hypocrea* spp. deducing mature peptide pheromones was not possible.
